# Radiation Exposure During Invasive Cardiovascular Procedures: Portable Shielding System Versus Standard Lead Aprons

**DOI:** 10.7759/cureus.68108

**Published:** 2024-08-29

**Authors:** Husam A Noor, Noof Althawadi, Zaina Noor, Nouf AlAnsari, Tarique S Chachar, Sara Al Raisi, Nooraldaem Yousif

**Affiliations:** 1 Cardiology Department, Mohammed Bin Khalifa Specialist Cardiac Centre (MKCC), Awali, BHR

**Keywords:** catheterization, radiation, lead apron, apron, lead, rampart

## Abstract

Introduction

Significant progress in the field of interventional cardiology has led to a rise in percutaneous procedures and an increase in the risk of radiation exposure at the workplace. Staff health has been put at risk due to the limitations of conventional radiation protective techniques. Innovative methods, such as RAMPART, have promising prospects for enhancing radiation safety. The purpose of this study was to evaluate RAMPART’s effectiveness and practicality in comparison to conventional protective techniques with a lead apron and shield (LAS) during cardiac interventional procedures.

Method

One hundred elective cardiac procedures were enrolled in this prospective single-center research study. Two groups were formed from the participants: standard protection (group A) and RAMPAT system (group B). Real-time dosimeters were used to track the radiation dosage, dosage reduction factor, dosage reduction percentage, and likelihood of exceeding the limit, which were included in the data. Proceduralists were urged to use different strategies to reduce exposure. The study was approved by an ethical committee and ran from June 2023 to August 2023.

Results

When comparing the RAMPART group to the conventional protection group, neck-level radiation exposure was considerably lower for all workers. There were no notable variations in the exposure of the waist. The RAMPART group was shown to be superior in minimizing radiation exposure, as evidenced by dose reduction metrics. The groups had comparable procedural characteristics.

Conclusion

Compared to conventional LAS, the RAMPART system dramatically reduces radiation exposure to the entire body.

## Introduction

The interventional cardiology field has advanced significantly in recent years. With many procedures currently performed percutaneously, there is a higher chance of occupational radiation exposure for those working in catheterization labs. The adverse effects of occupational radiation exposure are well recognized. These include increased cases of cataracts, skin reactions, and other types of malignancies, including those affecting the thyroid glands and the brain [1,2].

Historically, radiation protection during cardiac catheterization procedures has been achieved using architectural shielding and personal protective equipment. These include the standard requirements of lead aprons (one or two pieces) and thyroid collars, with the optional use of lead acrylic face masks, eyewear, skullcaps, and arm shields [3]. Nonetheless, recent reports showed a rise in various medical conditions, which could be attributed to inadequate protection of vital regions using standard radiation protection methods, including left-sided brain cancer as well as breast cancer [4]. Nonetheless, recent reports showed a rise in various medical conditions, which could be attributed to inadequate protection of vital regions using standard radiation protection methods, including left-sided brain cancer as well as breast cancer [5]. Additionally, the conventional method of radiation protection can lead to various orthopedic conditions due to the extended weight-bearing effect of lead aprons [6,7].

Recently, novel portable radiation protection systems, including RAMPART M1128 System, Protego Radiation Protection System, and Zero-Gravity System, have become available, aiming to offer better protection from scattered radiation and, therefore, mitigate to a certain extent the risks and health concerns associated with occupational radiation exposure, as well as reduce the burden of lead apron and its associated orthopedic injuries [7]. While the utility and adoption of these systems remain limited, initial reports with regard to their efficacy in reducing procedural radiation doses are promising [8].

In the present study, we sought to evaluate the feasibility and efficacy of using the RAMPART M1128 System during cardiac interventional procedures in a single center and to compare that with the current standard radiation protection method.

## Materials and methods

Methods

Study Design

This prospective single-center, two-arm study was conducted between June 2023 and August 2023. A total of 100 elective cardiac procedures were recruited into the study.

Procedures were performed in a catheterization lab equipped with a bi-plane C-arm Philips system using one of the two following methods of radiation protection: standard protection method (group A, N = 50) or portable shielding system (RAMPART M1128 System) (group B, N = 50). 

The radiation dose was monitored using real-time dosimeter readings (i3 RaySafe dosimetry system) from two different body levels (neck and waist level). A total of seven dosimeters were assigned. Two dosimeters were assigned to each of the operators, assistants, and radiographers. 

An additional dosimeter was placed under the patient’s chest (at the level of the sternum) as a control badge. The following measurements were then derived from real-time dosimeter readings: (1) dose reduction factor (DRF) calculated by dividing the average radiation recorded on the operator’s dosimeter by the radiation recorded on the control badge, (2) dose reduction percentage (DRP) calculated by subtracting 100 from DRF and multiplying by 100, and (3) probability of exceeding the limit calculated by subtracting the average DRF from 100. Proceduralists in both groups were encouraged to minimize radiation exposure as much as possible by closing any potential draft areas, closing gaps, using pulsed fluoroscopy and collimation, and using a lower frame rate. Cases included in the study were all elective procedures and percutaneous coronary intervention (PCI), including chronic total occlusion (CTO). Emergency cases and other cardiac procedures, including structural and electrophysiology procedures, were excluded from the study.

Ethical Consideration

The study protocol was reviewed and approved by the Research Ethical Committee (Institutional Review Board) of the Mohammed Bin Khalifa Bin Salman Al-Khalifa Specialist Cardiac Center, Awali, Bahrain, bearing approval number CTD-RES-2023-006. Informed consent was obtained from all patients and the staff recruited into the study.

Standard Protection Method

In the standards protection arm (group A), operators used a conventional two-piece lead apron (skirt and vest), thyroid collar, and a ceiling-mounted drop-down lead shield. Wrap-around skirts and vests were 0.5 mm thick (1 mm in the frontal area of overlap), and the back panel was 0.25 mm pb. Thyroid shields were 0.5 mm thick. The drop-down lead shield was covered with a sterile plastic drape to ensure sterility. The dosimeter at the neck level was worn above the lead apron to reflect the dose of the exposed body parts, while the waist dosimeter was worn under the apron to reflect the shielded body parts.

RAMPART M1128 System

The RAMPART M1128 System (group B) utilized a fully adjustable and configurable, floor-supported, and portable shielding system as described previously (reference). In summary, it comprises two 22-mm thick acrylic panels, each equivalent to 1-mm thick lead, and two soft shielding on each panel. Each soft shielding is equivalent to 0.5-mm thick lead. Under the table, lead pieces (×2), in addition to one abdominal protector, were used as per company recommendation. A sterile plastic drape is used per procedure to ensure sterility. In the RAMPAT group, both dosimeters at neck and waist levels were worn above the lead apron. The system is placed on the right side of the patient, separating the patient’s head from the procedural field and the operators. The panels can be adjusted to fit the patient’s girth, various proceduralists’ heights, and procedural setups.

Statistical analysis

Data analyses were carried out using IBM SPSS Statistics, version 27.0 (IBM Corp., Armonk, NY) software. Categorical variables were summarized using frequency and percentage for each category. Continuous variables were expressed as mean or median and standard deviation. The normality of the distribution was assessed using Shapiro-Wilk’s normality. Comparison between the RAMPART and non-RAMPART groups and radiation measurements were performed using the Mann-Whitney and chi-square tests. For all the tests, an alpha of 0.05 was considered statistically significant.

## Results

In total, 100 participants who underwent invasive cardiac procedures between June 2023 and August 2023 were recruited into the study. The majority of study participants were men (74%). However, there was no significant difference in gender distribution between the two groups (54.1% of group A were men versus 45.9% in group B, p = 0.25) (Table 1). Moreover, baseline patients’ characteristics, including height and weight, were also similar in the two groups (Table 2). Additionally, there were no significant differences in procedural characteristics between the two groups, including total fluoroscopy time (TFT), total radiation time, or control badge dose. Both groups had a similar number of diagnostic procedures and PCIs. However, all CTO procedures were recruited into the RAMPART group.

**Table 1:** Baseline patient and procedural characteristics for both RAMPART and non-RAMPART groups PCI, percutaneous coronary intervention; CTO, chronic total occlusion; n, number of participants; SD, standard deviation

Variables	Groups		p-value
	RAMPART, n (%)	Non-RAMPART, n (%)	
Gender			
Male	40 (54.1%)	34 (45.9%)	0.254
Female	10 (38.5%)	16 (61.5%)	
Type of procedure			
Diagnostic	23 (46.0%)	30 (60.0%)	0.315
PCI	23 (46.0%)	20 (40.0%)	0.695
CTO	4 (8.0%)	0 (0.0%)	-

**Table 2:** Demographics and procedural characteristics for both RAMPART and non-RAMPART groups TFT, total fluoroscopy time; n, number of participants; SD, standard deviation

Variables	Groups		p-value
	RAMPART, n (%)	Non-RAMPART, n (%)	
	Mean ± SD	Mean ± SD	
Demographics			
Height (cm)	166.8 ± 7.9	165.4 ± 8.7	0.717
Weight (kg)	81.5 ± 15.0	84.6 ± 19.0	0.782
Procedural details			
TFT (min)	12.3 ± 12.9	8.8 ± 11.1	0.192
Total radiation (mGy)	804 ± 661	815 ± 859	0.901
Control badge (uSv)	559 ± 459	535 ± 662	0.384

Neck and waist radiation exposure

**Figure 1:**
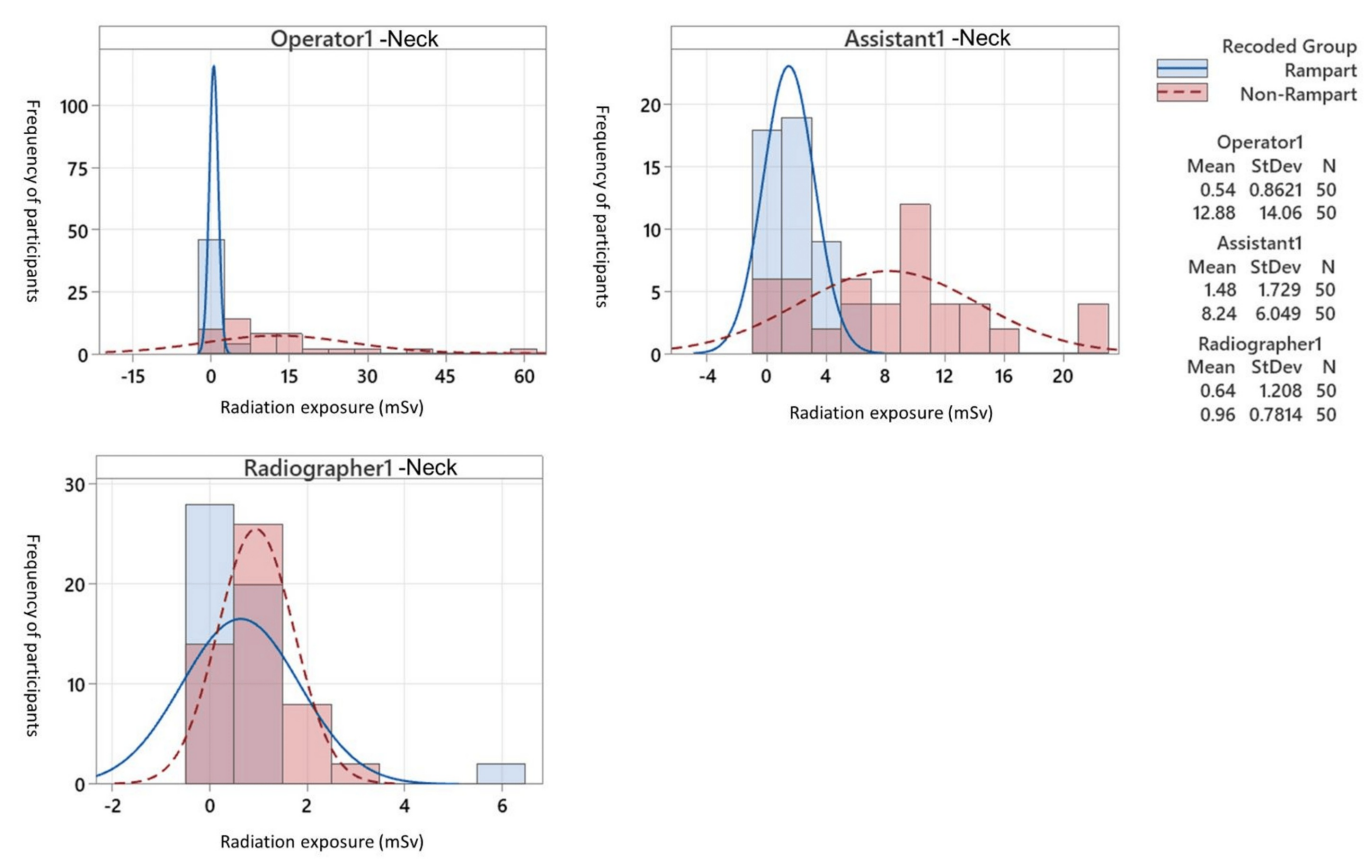
A graph demonstrating the distribution of radiation dose among operator, assistant, and radiographer from neck and waist level dosimeter

When comparing radiation exposure at neck level between the two groups, all staff in the RAMPART group, including the operator, assistant, and radiographer, had significantly lower radiation dose compared to their counterparts in the non-RAMPART group (0.5 ± 0.9, p < 0.0001; 1.5 ± 1.7, p < 0.0001; 0.6 ± 1.2, p = 0.002 for the operator, assistant, and radiographer, respectively). Nonetheless, there was no significant difference in waist exposure between the two groups across all staff (Table 3 and Figure 1).

**Table 3:** Radiation dose for the operator, assistant, and radiographer from neck and waist level dosimeter in both RAMPART and non-RAMPART groups SD, standard deviation

Variables	Groups		p-value
	RAMPART	Non-RAMPART	
	Mean ± SD	Mean ± SD	
Operator-neck (uSv)	0.5 ± 0.9	12.9 ± 14.1	<0.0001
Operator-waist (uSv)	1.0 ± 1.5	1.2 ± 2.4	0.395
Assistant-neck (uSv)	1.5 ± 1.7	8.2 ± 6.0	<0.0001
Assistant-waist (uSv)	0.8 ± 2.2	0.2 ± 0.7	0.118
Radiographer-neck (uSv)	0.6 ± 1.2	1.0 ± 0.8	0.002
Radiographer-waist (uSv)	0.2 ± 0.7	0.1 ± 0.3	0.941

Dose reduction parameters

The DRF (operator radiation dose relative to control badge dose) was lower in the RAMPART group across all staff (0.0019 ± 0.0025, p = 0.001; 0.0033 ± 0.0064, p = 0.001; 0.0019 ± 0.0067, p = 0.004 for the operator, assistant, and radiographer, respectively). This was also reflected in DRP where the RAMPART group had consistently higher values than the non-RAMPART group across all staff. Moreover, the probability of exceeding the limit was significantly lower in the RAMPART group for all staff (Table 4).

**Table 4:** Dose reduction parameters across the operator, assistant, and radiographer in both RAMPART and non-RAMPART groups DRF, dose reduction factor; DRP, dose reduction percentage; SD, standard deviation *Statistical significance

Variables	Groups		p-value
	RAMPART	Non-RAMPART	
	Mean ± SD	Mean ± SD	
DRF1 (operator)	0.0019 ± 0.0025	0.0227 ± 0.0285	0.0001*
DRF2 (assistant)	0.0033 ± 0.0064	0.0157 ± 0.0166	0.0001*
DRF3 (radiographer)	0.0019 ± 0.0067	0.0117 ± 0.0705	0.004*
DRP1 (operator)	-99.8095 ± 0.2515	-95.9308 ± 12.8864	0.0001*
DRP2 (assistant)	-99.6663 ± 0.6364	-98.4328 ± 1.6580	0.0001*
DRP3 (radiographer)	-99.8067 ± 0.6655	-98.8292 ± 7.0491	0.004*
Probability to exceed Limit	0.0024 ± 0.0030	0.0167 ± 0.0264	0.0001*

## Discussion

For this study, we assessed the RAMPART M1128 portable radiation shielding system and how well it reduced radiation exposure for the team in the cardiac catheterization laboratory during invasive cardiac procedures. Moreover, we compared its performance to that of the traditional radiation protection method. Overall, our findings indicate that utilizing the RAMPART portable radiation shielding system as a radiation protection technique during cardiac procedures is viable. Additionally, the RAMPART system proved to be more efficient in minimizing neck-level radiation exposure for all personnel than the conventional radiation protection method.

Radiation exposure in the interventional fields is associated with both direct and indirect occupational hazards. Despite the use of a lead apron and shields (LASs), prolonged and accumulative exposure to radiation imposes serious health concerns on all staff working in this environment.

Direct adverse effects from radiation exposure are divided into tissue and stochastic effects [1]. Deterministic injuries correspond to cell death secondary to radiation exposure exceeding the threshold, which could lead to cases of cataracts, skin erythema, and skin desquamation. On the other hand, exposure to cumulative radiation results in stochastic injuries, such as skin, thyroid gland, and brain malignancies. Increased incidents of left-sided brain cancer among workers in the radiation field could be attributed to inadequate protection of the head and neck areas when utilizing traditional protection methods [8,9]. Furthermore, extended exposure to radiation has been found to be associated with early atherosclerosis and vascular aging, mostly affecting the left carotid artery [10,11]. Overall, our results showed that the RAMPART system effectively reduced neck-level radiation exposure for all the staff compared to the traditional protection method. This may result in a reduced risk of health concerns associated with scattered radiation. Moreover, orthopedic conditions affecting the spine, hip, knee, and ankle that are not directly related to the effects of radiation but rather to the extended weight-bearing effect of lead aprons are likely to be reduced or eliminated by using the RAMPART system [11,12], which in turn can result in an improvement in primary operator productivity and reduce the number of missed workdays [13]. The findings of our study align with a recently published study that conducted a randomized comparison between the radiation exposure of RAMPART M1128 and the traditional lead-apron method [13,14].

There are several novel and lead-free radiation shielding systems that are commercially available in addition to the RAMPART system. These include the Protego Radiation Protection System, Zero-Gravity System, and Corindus CorPath robotic system [14]. The Protego system comprises a combination of radiation shields both above and below the table integrated with a flexible radiation-resistant drape. A single-center prospective study comparing the radiation exposure of physicians who used the Protego system to those who relied on standard protection demonstrated lower radiation exposure with the Protego system compared to standard protection at both thyroid and waist levels [10-14]. Nonetheless, the RAMPART system has the advantage of being portable compared to the nonportable Protego system. The Zero-Gravity System employs a 1.0-mm suspended lead body shield that magnetically connects with a vest worn by the primary operator, along with a 0.5-mm lead acrylic face shield. This system was found to decrease head-level physician radiation doses compared to the standard protection method [8,12]. However, it offers protection to the primary operator alone. Finally, the Corindus CorPath robotic system includes an articulating arm with a robotic drive and a single-use cassette that is connected to the patient table. Percutaneous coronary angiography is done through a touchscreen and joystick from a radiation-shielded interventional cockpit. A multi-center PRECISE study showed a median reduction in radiation exposure to the primary operator sitting at the cockpit of 95.2% compared to the traditional table position [15]. While this offers good radiation protection, procedural time is often prolonged due to the operator learning curve.

Limitations

Firstly, our study was a single-center study that was not randomized, and all CTO procedures were allocated to the RAMPART group. Despite this, a similar fluoroscopic system was used for all procedures, and the mean TFT was similar between the two groups. Differences in the experience and techniques of the operators performing the procedures may introduce variability in radiation exposure outcomes. Finally, the study was limited to measurements of radiation exposure to the neck and waist levels only, with the exclusion of other crucial body areas, including the head, axilla, forearm, and mid-tibia.

## Conclusions

Radiation exposure at the neck level was much lower when using the RAMPART M1128 radiation protection system during cardiac interventional procedures than when using the standard protection method, all without impeding workflow. This may lessen the risk of radiation exposure for those working in the catheterization laboratories and provide them with a sense of security in their workplace.
